# Potential and Limitation of HLA-Based Banking of Human Pluripotent Stem Cells for Cell Therapy

**DOI:** 10.1155/2014/518135

**Published:** 2014-07-09

**Authors:** Casimir de Rham, Jean Villard

**Affiliations:** Transplant Immunology Unit, Division of Immunology and Allergy and Division of Laboratory Medicine, University Hospital of Geneva, 4 rue Gabrielle Perret-Gentil, 1211 Geneva 14, Switzerland

## Abstract

Great hopes have been placed on human pluripotent stem (hPS) cells for therapy. Tissues or organs derived from hPS cells could be the best solution to cure many different human diseases, especially those who do not respond to standard medication or drugs, such as neurodegenerative diseases, heart failure, or diabetes. The origin of hPS is critical and the idea of creating a bank of well-characterized hPS cells has emerged, like the one that already exists for cord blood. However, the main obstacle in transplantation is the rejection of tissues or organ by the receiver, due to the three main immunological barriers: the human leukocyte antigen (HLA), the ABO blood group, and minor antigens. The problem could be circumvented by using autologous stem cells, like induced pluripotent stem (iPS) cells, derived directly from the patient. But iPS cells have limitations, especially regarding the disease of the recipient and possible difficulties to handle or prepare autologous iPS cells. Finally, reaching standards of good clinical or manufacturing practices could be challenging. That is why well-characterized and universal hPS cells could be a better solution. In this review, we will discuss the interest and the feasibility to establish hPS cells bank, as well as some economics and ethical issues.

## 1. Introduction

Human pluripotent stem (hPS) cells are undifferentiated cells that are capable of indefinite self-renewal and that can be derived into any cell of the human body. This means that, in specific environments, these cells are capable of differentiation into the three germ layers: endoderm, mesoderm and exoderm. Three different methods are currently available to obtain hPS cells. The first method is through the isolation of the inner mass cells of a blastocyst [1]. Following a specific culture protocol, these cells will give rise to one of the three germ layers that can be derived into specific cells or tissues depending on the protocol. The second method, which implies nuclear transfer, has only been done in animal, whose best example is the sheep Dolly [2]. This technique, called somatic cell nuclear transfer (SCNT), consists in isolating a nucleus from a somatic cell and transferring it into an oocyte, which has been previously enucleated [3]. This type of technique is no longer possible, mainly due to ethical reason. This method is not authorized with human cells; moreover, oocytes possess mitochondrias from maternal origin, which can be the source of minor histocompatibility antigens and could induce an alloreactive immune reaction [4].

Finally, the third method to obtain hPS cells is the generation of human induced pluripotent stem (hiPS) cells, which are derived from somatic adults cells, initially from fibroblasts. These somatic cells are transfected with four specific transcription factors,* Oct-4*,* c-Myc*,* Sox-2*, and* Klf-4* [5], and become reprogrammed in cells with pluripotency properties.

The technologies to obtain hPS cells are relatively sophisticated, but quite easy to obtain a significant number of stem cells. Alternative approaches are under investigation to obtain hPS cells such as the use of adult stem cells or MUSE cells. Adult stem cells refer to undifferentiated cells, found among differentiated tissues or organs throughout the body. These cells are also known as somatic stem cells or as germline stem cells, meaning that they are both pluripotent and multipotent. One major inconvenient is the rarity of adult stem cells; they represent 1 out of 10,000 cells within a given tissue. Adult stem cells exist as niches in different tissues and organs such as bone marrow, umbilical cord blood, liver, skin, or blood [6]. They can also be found in other tissues, such as adipose tissue [7], the dental pulp [8], the mammary gland [9], the olfactory mucosa [10], and the neural tissue [11].

Recently, Kuroda et al. [12] isolated a specific subpopulation named multilineage-differentiating stress-enduring (MUSE) cells. These cells are double positive for SSEA-3 (a specific marker of pluripotency) and for CD105 (a mesenchymal marker). However, these MUSE cells are rare; the ratio is 1 out of 3000 cells in the human bone marrow. But when MUSE cells are cultured as single cell, they proliferate and form a cluster, which is identical to an embryoid body of ES cells. These clusters express different pluripotency markers (SSEA-4; Nanog, Oct3/4) and differentiate into the three germ layers [12, 13]. Currently, hPS cells derived from adult cells (iPS) are the most efficient source of hPS cells. Human iPS cells possess similar pluripotent characteristics when compared to hES cells, indefinite self-renewal and differentiation in any cell or tissue. Since the first publication of derivation of hiPS cells by Takahashi et al. [5], using fibroblasts, several other publications have demonstrated the derivation of hiPS cells from different cell types such as peripheral blood cells [14], hepatocytes [15], or neural stem cells [16].

The first advantage of hiPS is to avoid the ethical problem of hES cells, the destruction of the blastocyst necessary to obtain hES cells. The second advantage is to avoid rejection of transplanted cells or tissue derived from hiPS cells, because hiPS cells can be theoretically generated from the patient being the recipient of the transplantation. Although hES and hiPS cells have similar pluripotentiality, they are not identical. Recent publications demonstrated differences between these two types of pluripotent stem cells, at the genetic level [17, 18] as well as at the epigenetic level [19]. Moreover, the production of hiPS cells includes viral vector transduction technology. This is a very efficient method, but if the virus, used to reprogram the cells, integrates the genome, this may lead to insertional mutation. This problem can now be overcome by nonintegrative reprogramming vectors [20] or small molecules [21]. Moreover, the age of the donor could be a problem; age could reduce the capacity of reprogramming the cell and increase the risk of chromosomal instability [22].

Finally, due to regulation procedures and economic factors, production of cells, tissues, or organs derived from hiPS cells and prepared for each recipient who need transplantation is not conceivable.

Adult stem cells and MUSE cells could be an alternative and should be more investigated. Although these cells exist at a very low ratio, they possess interesting features. They are directly available from the patient, and they do not need to be reprogrammed or to support strong manipulations in order to express pluripotency characteristics, which may avoid genetic dysfunction during cell division.

## 2. Immune Response against Pluripotent Stem Cells

Human PS cells are highly expected to satisfy the demand in tissue transplantation. Moreover, it was thought that hPS cells possess immune-privileged properties [26]. This assumption was clearly wrong as demonstrated by many studies in animal models and by in vitro studies in human [27–30].

If hPS cells from a donor are transplanted into a genetically unrelated recipient, these cells will be rejected by the recipient's immune system. Human leukocyte antigen (HLA), blood groups, and minor antigens are the three immunogenic hurdles that have to be avoided or crossed for a successful transplantation. Autologous transplantation with adult stem cells would be the best approach but, as already indicated, several problem make this option unrealistic [31].

The main immunologic hurdle that has to be overcome between two individuals, who are genetically different, is the human leukocyte antigen (HLA). HLA genes are located on chromosome 6 and represent the most polymorphic system in the human genome. They are divided into 2 groups of antigens, HLA class I or HLA-I (HLA-A, HLA-B, and HLA-C) expressed by nearly all nucleated cells and HLA class II or HLA-II (HLA-DR, HLA-DP, and HLA-DQ), which are expressed by some specific cells, such as dendritic or B cells.

The HLA system is the most polymorphic locus with almost 10,000 HLA-I and -II alleles, (http://hla.alleles.org). Therefore, the HLA typing of hPS cells of the donor and of the patient is mandatory to define any HLA mismatch between both donor and recipient.

CD8^+^T lymphocytes and natural killer (NK) cells are the two main effector cells that are able to target allogenic hPS cells and their derivatives. The killing mechanism of CD8^+^T-cells is mediated by the presentation of the antigen to the T-cell receptor, and NK cells kill the target cells expressing low level or no HLA-I. Because it has been demonstrated that hPS cells expressed low level of HLA-I [26, 32], these cells are therefore excellent targets for NK cells [33]. The progenitors and the mature cells derived from hPS cells will be expressed HLA-I and will be therefore excellent targets for CD8^+^T-cells. Consequently, immunosuppressive treatments are mandatory in protocols of transplantation to reduce the risk of rejection.

In solid organ transplantation, many studies have demonstrated the importance of HLA-A, HLA-B, and HLA-DR for the long-term graft survival in addition to immunosuppressive drugs. Although important progresses have been made within the immunosuppressive regimen, HLA-A, HLA-B, and HLA-DR matching, even at low resolution level, remain associated with better long-term graft outcome [34, 35]. Matching for these loci not only reduces the allograft rejection but also diminishes the use of immunosuppressive drugs [36, 37].

In addition to T and NK cells capacity to induce rejection, the presence of preformed antibodies directed against the ABO blood group (anti-ABO antibodies) could be a problem. Recipients with anti-HLA antibodies could theoretically reject hPS cells, or their derivatives expressing HLA antigens. It is not really clear if such antibodies would be harmful, but the most reasonable approach would be to perform transplantation with compatible ABO blood groups and to avoid the presence of anti-HLA antibody. In very specific situations of highly immunized recipients, plasma exchange, which reduces the level of anti-ABO or anti-HLA antibody, could be envisaged.

Matching hPS cells and recipients for HLA would be an endless work, due to the countless phenotype possibilities. One possibility to minimize this problem consists in using hPS cells, which are homozygote for common HLA haplotype from blood group O donors (universal donor).

## 3. Haplobanking of hPS Cells

The concept of a library or a bank to store cells and to use them for medical purpose is not new. This concept already exists for cord blood. Cord blood is a human tissue made of hematopoietic stem cells used for hematopoietic stem cell transplantation mainly in children but also in adults [38]. Cord blood possesses a large proportion of CD34^+^ cells, which are the source of all hematopoietic cell lineages. Cord blood is obtained, just after birth, from the umbilical cord and can be stored in liquid nitrogen for several years, for further therapeutic use. For successful hematopoietic stem cells transplantation, a large diversity of HLA alleles is necessary to find the best HLA match with the recipient. A similar approach could be envisaged with hiPS cells.

As already indicated, autologous iPS cells would be the best option to avoid the alloreaction, but obtaining such cells for each patient would be expensive, time consuming, and very difficult to produce.

The establishment of a hiPS cell bank would include most of the HLA diversity to have the best HLA compatibility with the recipient. Due to HLA diversity, this option would represent a bank with several millions of hiPS cells, which is not feasible.

Another possibility is a bank of HLA homozygous cell lines. It is possible to acquire homozygote cells, either by fertilizing donor gamete selected from paired volunteers, who possess shared haplotypes, or by SNCT. But using these techniques will raise major ethical and practical challenges [39]. The establishment of a clinical grade bank of homozygous cell lines, which can be expanded and differentiated for a large number of patients, would be more coherent [25].

Homozygous cell bank of hiPS cells would be more feasible compared to hES cells bank, since hiPS cells can be obtained from different tissues or from the patient himself. Ethical issue is a big concern and screening thousands of frozen embryo, in order to obtain hES cells, with unknown HLA at the time of the isolation of the inner mass cells would represent a massive work, almost impossible to envisage [40]. Homozygosis is not frequent and the question of the number of hPS cell lines that should be generated in order to cover a maximum number of individuals is critical.

**Figure 1:**
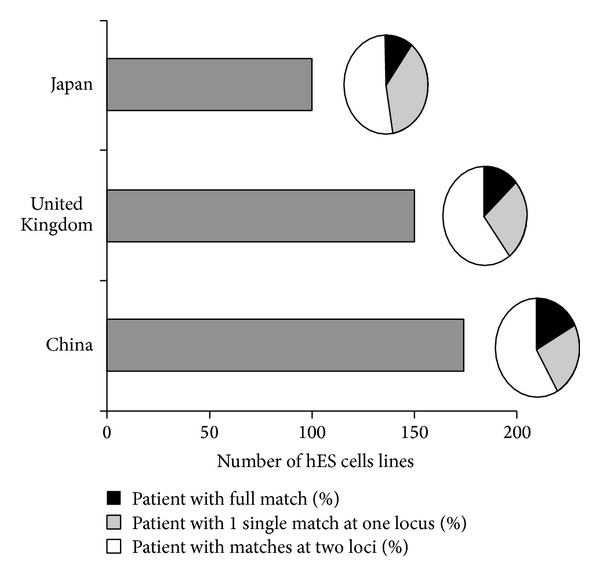
Hypothetical number of hPS cell lines haploidentical to a given population. Grey bars (left) represent the estimated number of homozygous hPS cell lines to achieve a full match (black; HLA-A, B and DR only) or two-match compatibility (white) with a given population (adapted from [23, 24, 39]).

Taylor et al. [39] and Nakajima et al. [23] both answered this question for their own countries, United Kingdom and Japan, respectively. Nakajima et al. calculated that if a bank possesses hPS cell lines from 100 randomly healthy selected donated embryo, 19% of patients were expected to find at least one hPS cell line with a complete matching for HLA-A, HLA-B, and HLA-DR in the Japanese population. Moreover, with the same number of embryo, the percentage of a single mismatch at one locus increases to 67% and even to 97% with a single mismatch at two loci. Taylor et al. showed that, in the UK, 150 donors would cover 18.5% of the population with a full match. Interestingly, using this number of 150 donors would also cover 21% of the Japanese population with a full match. The reason may be that the Japanese population possesses a more homogeneous HLA compared to the UK population [41] (Figure 1). As described, these 2 studies show results for a well-defined population. The frequency of conserved HLA haplotype in a given population is more complex when ethnical groups are mixed. In the 2 previous studies, the majority of the individuals were from European or from Japanese origin [42–45]. Of note, the rate of homozygosity at the HLA locus in a Caucasian population is low (1.1%) [46]. Whatever the source of hPS cells (embryonic, reprogrammed somatic cells…), this low frequency means that a large number of individuals should be screened to have homozygosity and sufficient haplotype to be representative of a given population in terms of HLA diversity. Interestingly, Lin et al. [24] did the same study with the Chinese population. They analyzed 188 hES cell lines from the Chinese Marrow Donor Program (CMDP) and their results were similar to those obtained by Taylor and Nakajima (Figure 1). Fraga et al. [47] characterized 22 human embryonic cells and they observed that they would match only with 0.011% of the Brazilian population. This demonstrates the high diversity in Brazilian population compared to the more homogenous Japanese population.

All these studies considered HLA-A, -B and -DR typing at low resolution. Haplobanking of hPS cell lines at high resolution level signify a much bigger number of cells and is probably not feasible. Therefore, even if a well-characterized haplobanking of hPS cells would be available in the near future, the transplantation would be performed between genetically unrelated donor and recipient. But the use of long-term immunosuppressive drugs for the recipients is still mandatory.

## 4. Practical Considerations for hPS Banks

The establishment of international rules for hPS cells haplobanking should be able to respond to practical points like the purpose of utilization, the financial aspects, the intellectual properties, and ethical rules. Three types of hPS cell banks could be listed: the public, the institutional, and the commercial bank. Public banks, such as the United Kingdom Stem Cell Bank, aim to be a source and a deposit of well-characterized hPS cells, with clear ethical rules. These hPS cell lines are for research and clinical purposes and conform to the British law [48]. The United States National Stem Cell Bank, established by the National Institute of Health (NIH), has the same policy. It stores, characterizes, and distributes the 21 hES cell lines listed on the NIH registry. Notice that these 21 hES cell lines are the only hES cell lines that can be used for research with federal funding [49]. Every scientist could have access to such a bank.

Institutional banks are established by local institutions such as universities, where hPS cell lines have been generated and stored locally. Such hPS cells are used for the institution research program or can be part of collaboration between universities [49]. The cost of public and institutional banks is covered by public funding (state, research institutes…) and nonprofit private foundations.

Commercial banks are private industries, which collect and/or generate hPS cell lines to provide them for research or clinical trials against money. The hPS cells belong to the bank and, thanks to the large financial resources of these industries, they are able to create and support high-quality and clinical grade hPS. For example, Advanced Cell Technology (ACT) is a private biotechnology company, which has its own hPS cell lines for research projects, but also takes part in clinical trials, such as blood vessel repair trial [49].

The intellectual property could be a complex problem. If an important discovery is made with one of the hPS cell lines provided by any bank, whom would the intellectual property rights belong to? To the bank, the researcher, or the institute where the research is done? The bank status is critical but probably it should avoid any type of intellectual properties. This property should be given to the researcher, or the institute where the research is done [49]. Finally, whatever the status of the bank, it must fill ethical rules and laws accepted by a majority of countries. hPS cells established or collected in one country could be used by researchers from other countries with the total respect of the legislation of all countries.

HiPS cells are the best (the only serious) candidates for hPS cells haplobanking. These hiPS cells would be generated from healthy volunteers with blood group O, to reduce the potential risk of alloimmune reaction mediated by anti-ABO agglutinins [45].

HiPS cells from any bank, used for medical application, must be generated following specific protocols such as avoidance of any animal product. They must fill good clinical practice conditions and the working and storing conditions must fill high quality standard confirmed by detailed protocols. Quality control surveillance should be part of the followup. However, hiPS cell banks for research only could be produced with lower standards.

In summary, a bank of HLA compatible hiPS cells that covers a wide number of individuals will be difficult to establish due to the HLA diversity. Haplobanking of hiPS will certainly reduce the number of cell lines and several banks that cover the various world populations would be the best option according to this HLA diversity. However, if HLA is the major genetic difference that impacts the rejection of allogenic transplanted tissue, the role of minor antigens in the rejection process should not be underestimated. Therefore, we believe that any transplantation of hiPS cells coming from genetically unrelated individual should signify the mandatory use of immunosuppressive drugs. The effort to establish a haplobanking of iPS cells is huge and a critical review of all arguments procontra should be carefully evaluated before starting such a project. From a purely immunological point of view it is probably not worth doing it.
